# High-Frequency Cochlear Amplifier Dysfunction: A Dominating Contribution to the Cognitive-Ear Link

**DOI:** 10.3389/fnagi.2021.767570

**Published:** 2022-01-06

**Authors:** Yao Wang, Xiao Li, Fuxin Ren, Siqi Liu, Wen Ma, Yue Zhang, Zhihang Qi, Jing Yang, Honghao Li, Xinxing Fu, Huiquan Wang, Fei Gao

**Affiliations:** ^1^School of Life Sciences, Tiangong University, Tianjin, China; ^2^School of Precision Instruments and Optoelectronics Engineering, Tianjin University, Tianjin, China; ^3^Department of Radiology, Shandong Provincial Hospital Affiliated to Shandong First Medical University, Jinan, China; ^4^Department of Radiology, Shandong Provincial Hospital, Cheeloo College of Medicine, Shandong University, Jinan, China; ^5^Department of Otolaryngology, Jinan Central Hospital, Cheeloo College of Medicine, Shandong University, Jinan, China; ^6^School of Electrical and Electronic Engineering, Tiangong University, Tianjin, China; ^7^Department of Neurology, Shandong Provincial Hospital Affiliated to Shandong First Medical University, Jinan, China; ^8^Beijing Institute of Otolaryngology, Otolaryngology-Head and Neck Surgery, Beijing Tongren Hospital, Capital Medical University, Beijing, China; ^9^Medical School, The University of Western Australia, Crawley, WA, Australia

**Keywords:** presbycusis, cognitive decline, high-frequency cochlear amplifier dysfunction, risk factor, cognitive-ear link

## Abstract

**Objective:** The objective of this study was to investigate the role of the high-frequency cochlear dysfunction in the cognitive-ear link.

**Methods:** Seventy-four presbycusis patients (PC group) and seventy-one age-, sex-, and education-level matched normal hearing controls (NH group) were recruited in this study. Participants underwent a battery of cognitive tests estimated by Montreal Cognitive Assessment (MoCA), Stroop Color-Word Interference Test (Stroop), Symbol Digit Modalities Test (SDMT), Auditory Verbal Learning Test (AVLT), and Trail-Making Test (TMT-A and B), as well as auditory tests including distortion product otoacoustic emission (DPOAE), pure tone (PT) thresholds, and speech reception thresholds (SRT). Data were analyzed using the factor analysis, partial correlation analysis, multiple linear regression models, and mediation models.

**Results:** Distortion product otoacoustic emission detection amplitudes and PT thresholds performed worse gradually from low to high frequencies in both the NH and PC groups. High-frequency DPOAE (H-DPOAE) was significantly correlated with cognitive domains in the PC group (AVLT: *r* = 0.30, *p* = 0.04; SDMT: *r* = 0.36, *p* = 0.01; Stroop: *r* = –0.32, *p* = 0.03; TMT-A: *r* = –0.40, *p* = 0.005; TMT-B: *r* = –0.34, *p* = 0.02). Multiple linear regression models showed that H-DPOAE predicted cognitive impairment effectively for aspects of memory (*R*^2^ = 0.27, 95% CI, 0.03 to 1.55), attention (*R*^2^ = 0.32, 95% CI, –6.18 to –0.40), processing speed (*R*^2^ = 0.37, 95% CI, 0.20 to 1.64), and executive function (TMT-A: *R*^2^ = 0.34, 95% CI, –5.52 to 1.03; TMT-B: *R*^2^ = 0.29, 95% CI, –11.30 to –1.12). H-DPOAE directly affected cognition and fully mediated the relationship between pure tone average (PTA)/SRT and cognitive test scores, excluding MoCA.

**Conclusion:** This study has demonstrated that the high-frequency cochlear amplifier dysfunction has a direct predictive effect on the cognitive decline and makes a large contribution to the cognitive-ear link.

## Introduction

Presbycusis (PC), as the high-incidence chronic disease in the elderly (Lin et al., 2011c), is associated with cognitive decline and dementia (Wayne and Johnsrude, 2015; Livingston et al., 2017, 2020; Loughrey et al., 2018; Jafari et al., 2019; Nixon et al., 2019; Brenowitz et al., 2020; Slade et al., 2020). Owing to the treatability of hearing loss, it has been considered as the most modifiable risk factor potentially in strategies for dementia prevention (Albers et al., 2015; Livingston et al., 2017, 2020; Loughrey et al., 2018; Chern and Golub, 2019).

Recent evidence for a link between cognitive impairment and hearing loss is based on diverse auditory assessments, including the pure tone average (PTA) (Lin et al., 2013; Harrison Bush et al., 2015; Croll et al., 2020), number of distortion product otoacoustic emissions (DPOAE) detected (Belkhiria et al., 2019, 2020), speech reception thresholds (SRT) (Castiglione et al., 2019; Ren et al., 2019), etc. In general, these findings imply an underlying significant interaction within the cognitive-ear link, which suggests the potential of correcting the hearing loss to achieve a better cognitive function in elder adults (Panza et al., 2018). In other words, early recognition of PC can be used to screen high-risk groups for cognitive impairment. Actually, PC involves the degeneration of peripheral and central auditory pathway (Keithley, 2020); thus, it is hard to distinguish the influences of peripheral from the central dysfunction (Gates and Mills, 2005). Most central PC cases with reduced speech discrimination also show a loss of outer hair cells (OHCs), which are helped by the cochlear amplifier to result in remarkable sound sensitivity and frequency specificity (Dallos and Evans, 1995; Gates and Mills, 2005). Even though PC most likely results from the degenerative changes of both OHCs and inner hair cells (IHCs) inevitably (Merchant and Nadol, 2010; Wu et al., 2020), whether dysfunction of OHCs or IHCs makes a great sense to the cognitive-ear link in PC remains unclear.

Considering that PC generally presents as degradation of the higher frequencies and progresses to degradation of the lower frequencies with time (Chern and Golub, 2019), we aimed to identify the role of the high-frequency cochlear dysfunction in the cognitive-ear link. In this study, the cochlear amplifier dysfunction was quantified as the DPOAE-detected amplitude, which is objective, frequency-specific, and sensitive to subtle changes in OHCs (Arnold et al., 1999; Prieve et al., 2020). We have conducted multiple-frequency band and modality of hearing assessment and hypothesized that the high-frequency cochlear amplifier dysfunction plays a dominant role in the cognitive-ear link.

## Materials and Methods

### Study Design and Participants

This study was approved by the Institutional Review Board of Shandong University (approval no. 2016-KY-059) and followed the Strengthening the Reporting of Observational Studies in Epidemiology (STROBE) reporting guideline. Each participant signed the informed written consent and received the payment.

The participants were native Mandarin Chinese speakers, right-handed (Jancke, 1996), and had no history of psychiatric or neurological diseases. Seventy-four PC patients (PC group) from the Department of Otolaryngology at the local hospital between March 2017 and April 2020 were recruited. Hearing loss has been defined as a PTA (range: 0.5–4 kHz) of the better ear ≥ 20 decibels hearing level (dB HL) (Stevens et al., 2013; World health organization, 2021). The exclusion criteria were (1) conductive hearing loss, asymmetric hearing loss, middle ear dysfunction, or tinnitus; (2) have used hearing aid, otologic surgery, ototoxic drug therapy, head trauma, or noise exposure; and (3) causes of sensorineural hearing loss other than PC. Seventy-one normal hearing controls (NH group) from the local community with the PTA less than 20 dB HL in both ears matched in age-, sex-, and education-level were recruited. Detailed inclusion and exclusion criteria for participants are shown in Supplementary Table 1.

### Auditory Assessments

All auditory evaluations were carried out in a sound-attenuating booth. Participants were assessed using a GSI Tympstar to confirm the optimal middle ear condition. Based on the results of the auditory assessment, the optimal ear was selected for the further analysis.

The pure tone (PT) audiometry threshold was assessed *via* a clinical audiometer (GSI AudioStar Pro, Grason-Stadler, Eden Prairie, MN, United States) coupled with TDH-50P telephonic headphones for each ear separately at frequencies of 0.125, 0.25, 0.5, 1, 2, 4, and 8 kHz. An extended PTA, including the 8 kHz threshold (PT threshold averages at frequencies of 0.5, 1, 2, 4, and 8 kHz), was also analyzed since the PC patients had predominantly a high-frequency hearing loss.

The outer hair cell function was evaluated using the DPOAE (2*f*1-*f*2, *f*2/*f*1 = 1.22) (Gorga et al., 1997; Ozimek et al., 2006), a type of otoacoustic emission induced by two primary PTs with a certain frequency ratio (Abdala and Dhar, 2012). DPOAE was recorded with a clinical otoacoustic emission detector (SmartOAE, Intelligent Hearing Systems, Miami, FL, United States) with a miniature microphone (ER-2, Etymotic Research, Elk Grove Village, IL, United States) inserted into the ear canal. Stimulus levels of *f*1 and *f*2 were kept at 65- and 55-dB sound pressure level, respectively. The frequencies of DPOAE detection were 0.5, 0.75, 1, 1.5, 2, 3, 4, 6, and 8 kHz. DPOAE amplitudes were determined by a signal-to-noise ratio > 6 dB for each frequency. Speech detection was assessed using the SRT (Schlauch et al., 1996), which was tested by a clinical audiometer (GSI AudioStar Pro, Grason-Stadler, Eden Prairie, MN, United States) equipped with TDH-50P telephonic headphones. An automatic HOPE software was adopted to deliver and evaluate the spondee words. The test was performed according to the SRT guidelines recommended by the American Speech Hearing Association.

### Cognitive Assessments

All cognitive assessments were completed within 60 min by a specialist in a fixed order. The Montreal Cognitive Assessment (MoCA) (Nasreddine et al., 2005), Stroop Color-Word Interference Test (Stroop) (Savitz and Jansen, 2003), Auditory Verbal Learning Test (AVLT, Chinese version) (Zhao et al., 2012), Symbol Digit Modalities Test (SDMT) (Benedict and Zivadinov, 2011), and Trail-Making Test (TMT) (Sanchez-Cubillo et al., 2009) were assessed for aspects of general cognitive function, attention, learning and memory, information processing speed, and executive function, respectively. The TMT consists of A and B; participants are required to draw sequential lines connecting 25 encircled numbers in TMT-A and draw a line between circles and squares alternately while connecting numbers in TMT-B. For each part of TMT, the final completion time is recorded as the test result (Wei et al., 2018).

### Statistical Analysis

The group differences in age, education, auditory, and cognitive tests were assessed by two-tailed *t*-test; the differences between PC and NH group in sex, hypertension, diabetes, hyperlipidemia, smoking, and bibulosity were evaluated by the chi-square test. Partial correlation analyses were employed to explore the relationships between cognitive and auditory functions in each group and the overall participant sample.

The PT thresholds of each frequency point were divided into low- and high-frequency bands by using the factor analysis with principal components extraction and Varimax rotation (Eckert et al., 2012). According to the component weights of the matrix after rotation (Supplementary Table 2), the PT thresholds of 0.125, 0.25, 0.5, and 1 kHz were loaded onto component 1 (low frequency), while the PT thresholds of 2, 4, and 8 kHz were loaded onto component 2 (high frequency). As the mean amplitude of DPOAE of all participants was positively correlated with the mean threshold value of PT (*r* = –0.488, *p* < 0.001), DPOAE amplitudes were divided into low and high frequencies according to components 1 and 2 of the PT thresholds. Subsequently, the partial correlation analysis was used to investigate the extent to which low-/high-frequency PT and DPOAE correlated with the cognitive function in the PC group, NH group, and all participants.

The multiple linear regression analysis was adopted to evaluate the influence of control variables and hearing function (high-frequency DPOAE, PTA, extended PTA, and SRT) on the global cognitive status and cognitive aspects. The controlled variables and auditory assessment measures were the independent variables, while the cognitive test scores were the dependent variables. The models with high-frequency DPOAE (H-DPOAE), PTA and extended PTA, and SRT as the independent variables are denoted as models 1, 2, 3, and 4 respectively. The degree of prediction is indicated by *R*^2^.

To identify the role of high-frequency cochlear amplifier dysfunction in the cognitive-ear link, mediation was tested by bootstrapping analyses in the SPSS software using two steps in all participants, namely, (1) the mediating effect of H-DPOAE on PTA/SRT and cognition and (2) the mediating effect of PTA/SRT on H-DPOAE and cognition. In all above analyses, the effects of age, sex, education, hypertension, diabetes, hyperlipidemia, smoking, and bibulosity were controlled. Data analyses were conducted using the SPSS 21.0 software.

## Results

### Demographics and Clinical Characteristics

**FIGURE 1:**
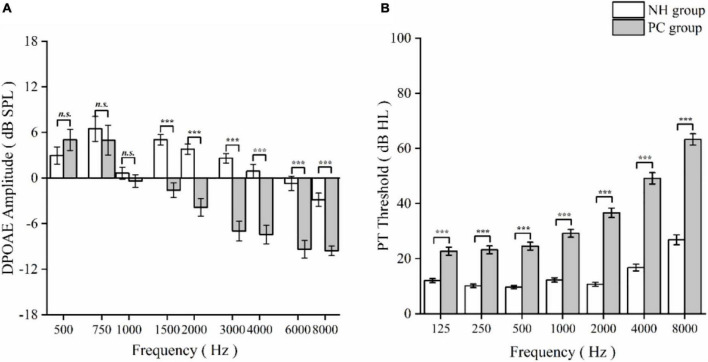
Comparison of distortion product otoacoustic emission (DPOAE) detection amplitudes and pure threshold (PT) thresholds between normal hearing (NH) and presbycusis (PC) groups as a function of frequency. **(A)** DPOAE detection amplitudes at different frequencies in NH and PC group. **(B)** PT thresholds at different frequencies in NH and PC groups. The data are presented as means ± standard error. Asterisk values indicate a statistically significant difference with a *p*-value < 0.05. *n. s.*, non-significant; ***p < 0.001.

Of the 74 PC patients [35 (7.3%) women; mean (SD) age, 62.74 (4.92) years] and 71 NH participants [43 (60.6%) women; 61.76 (4.62) years], no significant differences in age, sex, education, hypertension, diabetes, hyperlipidemia, smoking, or bibulosity were identified between the NH and PC groups (Table 1). The PC group showed significant disadvantages in DPOAE, PTA, PT, extended PTA, SRT (*p* < 0.001; Table 1), MoCA, AVLT, SDMT, Stroop, TMT-A, and TMT-B (*p* < 0.05; Table 1) compared to the NH group. The DPOAE detection amplitudes and PT thresholds worsened gradually from low to high frequencies in the PC group (Figure 1).

**TABLE 1:** Demographics and clinical data of participants.

Variable	NH group, No. (%)	PC group, No. (%)	All participants, No. (%)	*p-*value
	(*n* = 71)	(*n* = 74)	(*n* = 145)	NH vs. PC
Age, mean (SD), y	61.76 (4.62)	62.74 (4.92)	62.26 (4.79)	0.22
Education, mean (SD), y	11.73 (2.92)	10.78 (3.24)	11.25 (3.11)	0.07
Sex				0.12
Male	28 (39)	39 (53)	67 (46.2)	
Female	43 (61)	35 (47)	78 (53.8)	
Hypertension				0.30
Yes	22 (31)	29 (39)	51 (35.2)	
No	49 (69)	45 (61)	94 (64.8)	
Diabetes				0.49
Yes	7 (10)	10 (14)	17 (11.7)	
No	64 (90)	64 (86)	128 (88.3)	
Hyperlipidemia				0.54
Yes	9 (13)	7 (9)	16 (11)	
No	62 (87)	67 (91)	129 (89)	
Smoking				0.56
Yes	4 (6)	6 (8)	10 (6.9)	
No	67 (94)	68 (92)	135 (93.1)	
Bibulosity				0.74
Yes	3 (4)	4 (5)	7 (4.8)	
No	68 (96)	70 (95)	138 (95.2)	
DPOAE, mean (SD), y	1.95 (3.50)	–2.86 (5.89)	–0.51 (5.42)	< 0.001***
PTA, mean (SD), y	12.23 (4.31)	35.47 (10.62)	24.09 (14.22)	< 0.001***
PT, mean (SD), y	14.05 (4.72)	35.75 (9.75)	25.13 (13.32)	< 0.001***
EX-PTA, mean (SD), y	15.24 (5.53)	40.86 (10.60)	28.32 (15.40)	< 0.001***
SRT, mean (SD), y	12.50 (4.36)	35.76 (12.77)	24.37 (15.10)	< 0.001***
MoCA, mean (SD), y	25.89 (3.16)	23.58 (5.07)	24.71 (4.38)	0.003**
AVLT, mean (SD), y	53.97 (11.33)	45.89 (13.06)	49.85 (12.86)	< 0.001***
SDMT, mean (SD), y	31.38 (10.98)	25.59 (12.49)	28.43 (12.09)	0.004**
Stroop, mean (SD), y	138.08 (32.48)	152.14 (47.61)	145.26 (41.37)	0.04*
TMT-A, mean (SD), y	62.45 (25.27)	78.27 (37.50)	70.52 (32.96)	0.003**
TMT-B, mean (SD), y	167.72 (69.70)	206.61 (80.85)	187.57 (77.82)	0.002**

DPOAE, distortion product otoacoustic emission; PTA, pure tone average in four frequencies; PT, pure tone average in all frequencies; EX-PTA, extended PTA including 8 kHz, pure tone threshold averages at frequencies of 0.5, 1, 2, 4, and 8 kHz; SRT, speech reception threshold; MoCA, Montreal Cognitive Assessment; AVLT, Auditory Verbal Learning Test; SDMT, Symbol Digit Modalities Test; TMT, Trail-Making Test. The data are presented as means ± standard deviations. Asterisk values in bold indicates a statistically significant difference with a p-value < 0.05. *p < 0.05, **p < 0.01, and ***p < 0.001.

### Association Between Hearing Assessments and Cognition

**FIGURE 2:**
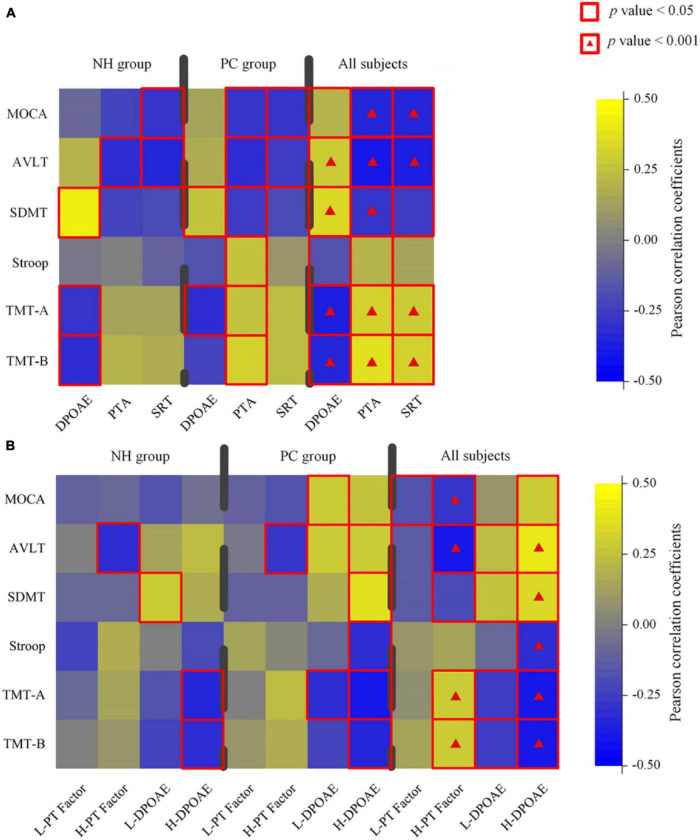
Characterization of the relationship between hearing loss and cognitive data in normal hearing (NH), presbycusis (PC) groups, and all participants. **(A)** A correlation between all-frequency hearing assessments and cognitive scores in different groups. **(B)** A correlation between low/high-frequency hearing assessments and cognitive scores in different groups. Color scale represents Pearson correlation coefficient. Red square alone or with a hash indicates statistical significance, respectively, at a threshold of *p* < 0.05 and *p* < 0.001. PTA, pure tone average in four frequencies; PT, pure tone average in all frequencies; SRT, speech reception threshold; L/H, low/high frequency; MoCA, Montreal Cognitive Assessment; AVLT, Auditory Verbal Learning Test; SDMT, Symbol Digit Modalities Test; TMT, Trail-Making Test.

The PTA was associated with cognitive assessments in both PC group and all participants (Figure 2A; detailed data: Table 2). DPOAE was correlated with cognitive assessments in all participants and was correlated with SDMT and TMT-A in the PC group. The extended PTA was associated with cognitive assessments in all participants and was correlated with MoCA, AVLT, and TMT-B in the PC group.

**TABLE 2:** The correlation relationship between audiological test and cognitive test.

Auditory	Group	Correlation	MOCA	AVLT	SDMT	Stroop	TMT-A	TMT-B
DPOAE	NH	*r*	–0.086	0.186	0.424**	–0.023	−−0.290*	−−0.314*
		*p*-value	0.50	0.15	**0.001**	0.86	**0.02**	**0.01**
	PC	*r*	0.148	0.183	0.253*	–0.175	−−0.307*	–0.232
		*p*-value	0.24	0.14	**0.04**	0.16	**0.01**	0.06
	All	*r*	0.190*	0.289***	0.347***	−−0.184*	−−0.361***	−−0.334***
		*p*-value	**0.03**	**< 0.001**	**< 0.001**	**0.03**	**< 0.001**	**< 0.001**
PTA	NH	*r*	–0.230	−−0.302*	–0.238	–0.004	0.130	0.197
		*p*-value	0.07	**0.02**	0.06	0.98	0.31	0.12
	PC	*r*	−−0.299*	−−0.308*	−−0.265*	0.248*	0.256*	0.303*
		*p*-value	**0.02**	**0.01**	**0.03**	**0.05**	**0.04**	**0.01**
	All	*r*	−−0.349***	−−0.395***	−−0.284***	0.208*	0.305***	0.359***
		*p*-value	**< 0.001**	**< 0.001**	**< 0.001**	**0.02**	**< 0.001**	**< 0.001**
EX-PTA	NH	*r*	–0.188	−−0.307*	–0.122	0.086	0.069	0.074
		*p*-value	0.13	**0.01**	0.32	0.48	0.58	0.55
	PC	*r*	−−0.246*	−−0.300*	–0.181	0.106	0.207	0.257*
		*p*-value	**0.04**	**0.01**	0.13	0.38	0.08	**0.03**
	All	*r*	−−0.313***	−−0.400***	−−0.241**	0.166*	0.286**	0.325***
		*p-*value	**< 0.001**	**< 0.001**	**0.004**	**0.048**	**0.001**	**< 0.001**
SRT	NH	*r*	−−0.272*	−−0.335**	–0.214	–0.115	0.150	0.160
		*p*-value	**0.03**	**0.007**	0.09	0.37	0.24	0.21
	PC	*r*	−−0.284*	−−0.246*	–0.210	0.091	0.219	0.221
		*p*-value	**0.02**	**0.05**	0.09	0.47	0.08	0.08
	All	*r*	−−0.351***	−−0.374***	−−0.260**	0.131	0.294***	0.327***
		*p-*value	**< 0.001**	**< 0.001**	**0.002**	0.13	**< 0.001**	**< 0.001**

Partial correlation analyses were used and controlled for age, sex, education degree, hypertension, diabetes, hyperlipidemia, smoking, and bibulosity. Asterisk values in bold indicate a statistically significant difference with a p-value < 0.05. *p < 0.05, **p < 0.01, and ***p < 0.001.

For high-frequency hearing loss, the high-frequency PT threshold factor was positively correlated with TMT-A and TMT-B and negatively correlated with MoCA, AVLT, and SDMT. In all participants, H-DPOAE was significantly associated with MoCA, AVLT, SDMT, Stroop, TMT-A, and TMT-B. In the PC group, H-DPOAE was associated with AVLT, SDMT, Stroop, TMT-A, and TMT-B significantly (Figure 2B; detailed data: Tables 3, 4).

**TABLE 3:** Relationship between low- and high-frequency PT thresholds factor and cognitive tests.

Cognition	NH group				PC group				All subjects			
	Low frequency		High frequency		Low frequency		High frequency		Low frequency		High frequency	
	*r*	*p-*value	*r*	*p-*value	*r*	*p-*value	*r*	*p-*value	*r*	*p-*value	*r*	*p-*value
MOCA	–0.120	0.35	–0.075	0.56	–0.111	0.37	–0.185	0.14	–0.174*	**0.04**	−−0.298***	**<0.001**
AVLT	0.004	0.98	–0.315*	**0.01**	–0.026	0.84	–0.297*	**0.02**	–0.140	0.10	−−0.390***	**<0.001**
SDMT	–0.094	0.47	–0.073	0.57	–0.106	0.40	–0.126	0.31	–0.153	0.08	−−0.206*	**0.02**
Stroop	–0.235	0.06	0.165	0.20	0.148	0.24	0.024	0.85	0.097	0.26	0.144	0.09
TMT–A	–0.094	0.46	0.157	0.22	–0.003	0.98	0.221	0.07	0.061	0.48	0.299***	**<0.001**
TMT–B	–0.013	0.92	0.082	0.52	0.091	0.47	0.170	0.17	0.146	0.09	0.293***	**<0.001**

Asterisk values in bold indicate a statistically significant difference with a p-value < 0.05. *p < 0.05 and ***p < 0.001. Stroop, Stroop Color-Word Interference Test.

**TABLE 4:** Relationship between low- and high-frequency DPOAE amplitudes and cognitive tests.

Cognition	NH group				PC group				All subjects			
	Low frequency		High frequency		Low frequency		High frequency		Low frequency		High frequency	
	*r*	*p-*value	*r*	*p-*value	*r*	*p-*value	*r*	*p-*value	*r*	*p-*value	*r*	*p-*value
MOCA	–0.174	0.12	–0.051	0.70	0.291*	**0.04**	0.256	0.08	0.099	0.30	0.294**	**0.001**
AVLT	0.130	0.34	0.232	0.07	0.275	0.06	0.299*	**0.04**	0.227*	**0.02**	0.390***	**<0.001**
SDMT	0.300*	**0.03**	0.181	0.16	0.183	0.21	0.358*	**0.01**	0.267**	**0.004**	0.336***	**<0.001**
Stroop	0.008	0.96	–0.200	0.12	–0.081	0.58	–0.324*	**0.03**	–0.092	0.33	–0.312***	**<0.001**
TMT-A	–0.176	0.20	–0.350*	**0.006**	–0.322*	**0.02**	–0.402**	**0.005**	–0.246**	**0.009**	–0.402***	**<0.001**
TMT-B	–0.220	0.01	–0.304*	**0.02**	–0.226	0.17	–0.344*	**0.02**	–0.244**	**0.009**	–0.405***	**<0.001**

Asterisk values in bold indicate a statistically significant difference with a p-value < 0.05. *p < 0.05, **p < 0.01, and ***p < 0.001.

### Regression Models of Auditory Function and Cognitive Assessment

The corresponding models with the various auditory variables are shown in Table 5. In all participants, the model of H-DPOAE with a higher correlation coefficient offered a better prediction value compared with PTA, extended PTA, and SRT for aspects of memory (0.25 vs. 0.22 vs. 0.23 vs. 0.20), attention (0.23 vs. 0.20 vs. 0.19), processing speed (0.31 vs. 0.29 vs. 0.27 vs. 0.28), and executive function (TMT-A: 0.31 vs. 0.21 vs. 0.20 vs. 0.20; TMT-B: 0.27 vs. 0.22 vs. 0.19 vs. 0.20). In the PC group, the correlation coefficients of the three models showed that H-DPOAE predicted cognitive impairment effectively for aspects of memory (*R*^2^ = 0.27, 95% CI, 0.03 to 1.55; *p* = 0.04), attention (*R*^2^ = 0.32, 95% CI, –6.18 to –0.40; *p* = 0.03), processing speed (*R*^2^ = 0.37, 95% CI, 0.20 to 1.64; *p* = 0.01), and executive function (*R*^2^ = 0.34, 95% CI, –5.52 to 1.03; *p* = 0.005 for TMT-A; *R*^2^ = 0.29, 95% CI, –11.30 to –1.12; *p* = 0.02 for TMT-B).

**TABLE 5:** Multivariate linear regression models with different auditory variables.

Cognition	Model I (H-DPOAE et al. * as independent variables)			Model II (PTA et al. * as independent variables)			Model III (EX-PTA et al. * as independent variables)			Model IV (SRT et al. * as independent variables)		
	β (95% CI)	*P-*value	*R* ^2^	β (95% CI)	*p-*value	*R* ^2^	β (95% CI)	*p-*value	*R* ^2^	β (95% CI)	*p-*value	*R* ^2^
**All subjects**												
MoCA	0.26 (0.07 to 0.29)	**0.001**	0.35	–0.31 (–0.14 to –0.05)	**<0.001**	0.37	–0.30 (–0.13 to –0.05)	**<0.001**	0.36	–0.31 (–0.13 to –0.05)	**<0.001**	0.37
AVLT	0.38 (0.44 to 1.13)	**<0.001**	0.25	–0.40 (–0.50 to –0.22)	**<0.001**	0.22	–0.41 (–0.47 to –0.21)	**<0.001**	0.23	–0.38 (–0.46 to –0.19)	**<0.001**	0.20
SDMT	0.31 (0.30 to 0.94)	**<0.001**	0.31	–0.26 (–0.35 to –0.09)	**0.001**	0.29	–0.23 (–0.30 to –0.06)	**0.003**	0.27	–0.24 (–0.31 to –0.07)	**0.002**	0.28
Stroop	–0.30 (–3.20 to –0.90)	**0.001**	0.23	0.20 (0.12 to 1.04)	**0.02**	0.20	0.16 (0.005 to –0.88)	**0.05**	0.19	N/A	0.13	0.18
TMT-A	–0.38 (–2.95 to –1.20)	**<0.001**	0.31	0.30 (0.32 to 1.05)	**<0.001**	0.21	0.28 (0.25 to 0.91)	**0.001**	0.20	0.29 (0.28 to 0.98)	**<0.001**	0.20
TMT-B	–0.39 (–7.38 to –3.03)	**<0.001**	0.27	0.36 (1.09 to 2.80)	**<0.001**	0.22	0.32 (0.81 to 2.42)	**<0.001**	0.19	0.33 (0.85 to 2.50)	**<0.001**	0.20
**PC group**												
MoCA	N/A	0.08	0.37	–0.26 (–0.22 to –0.03)	**0.02**	0.38	–0.24 (–0.21 to –0.02)	**0.03**	0.37	–0.25 (–0.18 to –0.02)	**0.02**	0.38
AVLT	0.30 (0.03 to 1.55)	**0.04**	0.27	–0.31 (–0.68 to –0.09)	**0.01**	0.17	–0.32 (–0.69 to –0.10)	**0.009**	0.18	–0.25 (–0.50 to –0.004)	**0.05**	0.14
SDMT	0.34 (0.20 to 1.64)	**0.01**	0.37	–0.25 (–0.56 to –0.03)	**0.03**	0.27	–0.19 (–0.50 to 0.04)	0.10	0.25	N/A	0.09	0.25
Stroop	–0.31 (–6.18 to –0.40)	**0.03**	0.32	0.23 (0.03 to 2.06)	**0.05**	0.26	0.13 (–0.46 to 1.61)	0.27	0.23	N/A	0.47	0.22
TMT-A	–0.40 (–5.52 to –1.03)	**0.005**	0.34	0.25 (0.05 to 1.74)	**0.04**	0.18	0.20 (–0.14 to 1.57)	0.10	0.16	N/A	0.08	0.16
TMT-B	–0.34 (–11.30 to –1.12)	**0.02**	0.29	0.29 (0.48 to 3.98)	**0.01**	0.24	0.24 (0.03 to 3.59)	**0.05**	0.21	N/A	0.08	0.20
**NH group**												
MoCA	N/A	0.70	0.25	N/A	0.07	0.33	–0.13 (–0.21 to 0.06)	0.27	0.31	–0.25 (–0.34 to –0.02)	**0.03**	0.35
AVLT	N/A	0.07	0.17	–0.30 (–1.44 to –0.15)	**0.02**	0.22	–0.37 (–1.26 to –0.24)	**0.004**	0.25	–0.34 (–1.52 to –0.25)	**0.01**	0.23
SDMT	N/A	0.16	0.24	N/A	0.06	0.31	–0.13 (–0.74 to 0.21)	0.27	0.29	N/A	0.09	0.31
Stroop	N/A	0.12	0.27	N/A	0.98	0.28	0.03 (–1.24 to 1.60)	0.80	0.28	N/A	0.37	0.29
TMT-A	–0.36 (–4.19 to –0.75)	**0.006**	0.28	N/A	0.31	0.17	0.10 (–0.72 to 1.65)	0.44	0.17	N/A	0.24	0.18
TMT-B	–0.31 (–10.78 to –1.08)	**0.02**	0.25	N/A	0.12	0.18	0.08 (–2.36 to 4.26)	0.57	0.15	N/A	0.21	0.17

*Other independent variables include age, sex, education degree, hypertension, diabetes, hyperlipidemia, smoking and bibulosity. The β weights indicate the standard deviation change in the outcome associated with auditory indicators. The 95% CI indicates the confidence interval or range of values across which β would be expected to occur 95% of the time. The change in R^2^ represents the variance of the contribution of auditory and control variables to cognitive variables. N/A, not available (regression model could not be establish). The bold values indicate a statistically significant correlation.

### Mediation Analysis Between Hearing and Cognition

**FIGURE 3:**
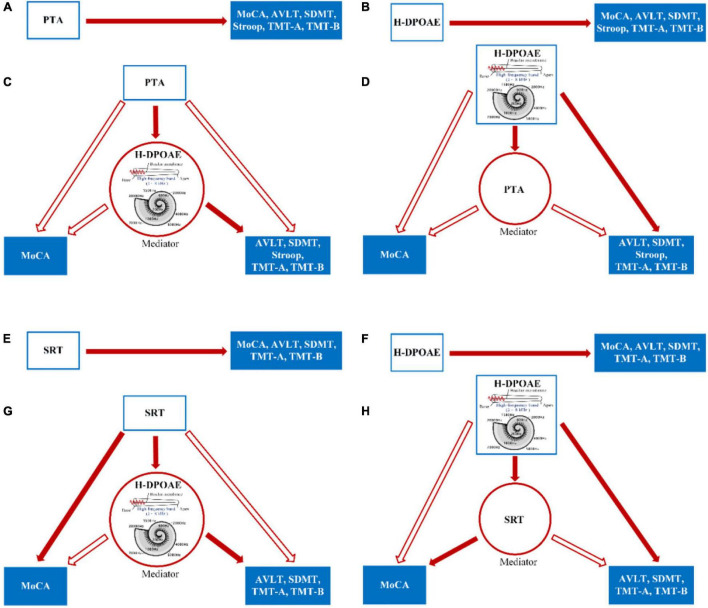
The cascading mediation effects between H-DPOAE, PTA/SRT, and cognitive tests in all participants. **(A)** Total effect of PTA predicting cognition. **(B)** Total effect of H-DPOAE predicting cognition. **(C)** Mediation by H-DPOAE between PTA and MoCA, AVLT, Stroop, TMT-A, and TMT-B. **(D)** Mediation by PTA between H-DPOAE and MoCA, AVLT, Stroop, TMT-A, and TMT-B. **(E)** Total effect of SRT predicting cognition. **(F)** Total effect of H-DPOAE predicting cognition. **(G)** Mediation by H-DPOAE between SRT and MoCA, AVLT, TMT-A, and TMT-B. **(H)** Mediation by SRT between H-DPOAE and MoCA, AVLT, TMT-A, and TMT-B. Red solid arrows indicate the significant paths resulting in mediation, and red hollow arrows indicate the insignificance of paths. Blue hollow boxes indicate independent variables, red hollow circles indicate mediators, and blue solid boxes indicate dependent variables. H-DPOAE, high-frequency DPOAE.

Figure 3 shows the cascading mediation effects of H-DPOAE on the relationship of PTA/SRT and cognitive tests in all participants. The mediation analysis characteristics are shown in Supplementary Figures 1, 2.

#### PTA/H-DPOAE—Cognition

The PTA showed a total indirect effect on all cognitive tests; however, there was no direct effect after controlling for H-DPOAE. H-DPOAE was not correlated with MoCA (β = 0.10, 95% CI, –0.05 to 0.25; *p* = 0.20), indicating the absence of a mediation effect of H-DPOAE between PTA and MoCA. An indirect effect of PTA on AVLT, SDMT, Stroop, TMT-A, and TMT-B was present. Therefore, H-DPOAE fully mediated the relationship between PTA and cognitive test scores, except for MoCA.

#### H-DPOAE/PTA—Cognition

The high-frequency DPOAE (H-DPOAE) showed a total indirect effect on all six cognitive test scores and a direct effect after controlling for PTA, excluding MoCA. PTA was not correlated with any cognitive test scores, indicating the absence of a mediation effect of PTA between H-DPOAE and cognition.

#### SRT/H-DPOAE—Cognition

The speech reception threshold showed a total indirect effect on all six cognitive test scores. The direct effect of SRT was significant on MoCA (β = –0.07, 95% CI, –0.13 to –0.003; *p* = 0.04) but not significant on the other five cognitive tests. H-DPOAE was correlated with AVLT, SDMT, TMT-A, and TMT-B but was not correlated with MoCA (β = 0.08, 95% CI, –0.06 to 0.22; *p* = 0.23). Therefore, H-DPOAE fully mediated the relationship between SRT and AVLT, SDMT, TMT-A, and TMT-B.

#### H-DPOAE/SRT—Cognition

The H-DPOAE showed a total indirect effect on all six cognitive test scores. The direct effect of H-DPOAE was significant on AVLT, SDMT, TMT-A, and TMT-B but not significant on MoCA (β = 0.08, 95% CI, –0.06 to 0.22; *p* = 0.23). SRT was correlated with MoCA (β = –0.07, 95% CI, –0.13 to –0.003; *p* = 0.04) but was not significantly correlated with the other five cognitive test scores. Therefore, SRT fully mediated the relationship between H-DPOAE and MoCA.

## Discussion

In this study, we measured multiple frequency bands and modalities of hearing indices and carried out multiple cognitive tests in participants with PC and normal hearing. Given the topographic organization of the cochlea, this study divided the DPOAE amplitudes and PT thresholds into low- and high-frequency bands. Interestingly, H-DPOAE was a better predictor of cognitive aspects than PTA and SRT in the PC group. For all participants, H-DPOAE fully mediated the relationship between PTA/SRT and cognitive domain in memory, attention, processing speed, and executive function.

Accumulating evidence supports a reliable association between hearing loss and cognition (Lin, 2011; Lin et al., 2011a,b, 2013; Wingfield and Peelle, 2012). This finding implies a hearing-cognitive interaction and the need to consider the entire ear-brain connection. However, whether the OHC dysfunction contributes to this association is still unknown. Our findings suggest an association between the cochlear amplifier dysfunction and cognitive decline in the NH group, PC group, and all participants. As far as we know, our study illustrates the relationship between hearing and cognitive function from multiple domains of auditory using both objective and subjective assessments for the first time. Previous research of the relationship between age-related hearing loss and cognitive decline has mainly focused on hearing assessed by PT thresholds (Harrison Bush et al., 2015; Chern and Golub, 2019; Croll et al., 2020). In our study, the association between hearing indicated by PTA and cognition was consistent with previous findings. Although PT thresholds rely on the optimal susceptibility of a few OHCs and IHCs, as well as their related eighth nerve fibers (Gates and Mills, 2005), the test still requires participant cooperation and does not reflect peripheral hearing objectively. That is why we used DPOAE to investigate the relationship between the cochlear and cognitive function. Only two previous studies have reported associations between the cognitive assessment and cochlear amplifier dysfunction assessed by the number of DPOAEs detected (Belkhiria et al., 2019, 2020). However, considering the clinical application of DPOAE-detected amplitudes, we adopted DPOAE as a quantitative assessment of cochlear function that is sensitive to subtle changes in OHCs (Arnold et al., 1999).

Interestingly, we observed that H-DPOAE was significantly correlated with cognitive domains in both the PC group and all participants. Furthermore, H-DPOAE predicted cognition better than PTA and SRT in terms of memory, attention, processing speed, and executive function. This may be because age-related hearing loss is characterized by the decline in hearing sensitivity from high to low frequencies (Kobrina et al., 2020). A recent study reported that extended high frequencies (8–20 kHz) may be a sensitive predictor of PC earlier in life when preventive methods can be effectively used (Motlagh Zadeh et al., 2019). In this study, the extended PTA was only associated with MoCA, AVLT, and TMT-B for the PC group. Thus, the extended PTA may not be an equivalent representation compared with H-DPOAE in terms of association between the high-frequency hearing loss and cognitive decline. In addition, extended high-frequency hearing can also influence DPOAE at lower frequencies. For example, 4–8 kHz DPOAE levels were correlated with PTA from 11.2–20 kHz significantly (Arnold et al., 1999). Therefore, H-DPOAE reflects the cochlear dysfunction in a more extensive frequency band range. Most importantly, high-frequency cochlear degeneration measured by H-DPOAE suggested earlier and more variable deterioration than indicated by behavior. As a concern regarding the significant relationship between the degree of cognitive deficit and hearing impairment (Taljaard et al., 2016), the use of H-DPOAE to predict cognitive impairment could be performed earlier in time.

A further novel result of the present study was that the cochlear amplifier dysfunction affected cognition directly and H-DPOAE fully mediated the relationship between PTA/SRT and cognitive test scores, excluding MoCA. However, PTA and SRT had significant direct effects on cognitive test scores, except MoCA, after controlling for H-DPOAE. These results suggest a dominant role of the high-frequency cochlear amplifier dysfunction in the cognitive-ear link. A recent quantitative microscopic analysis of inner ear damage in human patients revealed that PC is characterized by damage to inner ear sensory cells, and that the degree of hearing loss is closely predicted by the amount of hair cell loss (Wu et al., 2020). Additionally, OHC-based cochlear amplification at higher frequencies contributes more greatly to the association with cognition. These findings may explain why H-DPOAE is a key predictor of cognitive domain scores. As MoCA is a globally used cognitive screening tool that captures limited variability in a normally elderly population, it may potentially underestimate the true relationship between PC indicted by H-DPOAE and cognitive decline (Panza et al., 2018).

Several possibilities likely explained the association between the peripheral hearing capability and cognition observed in this study. Previous studies consider social isolation as a potential explanation for the link of PC and cognitive decline (Wayne and Johnsrude, 2015; Deal et al., 2017), while the positive association of hearing aid use on cognition in the prior study has been observed independent of social isolation or depression (Dawes et al., 2015). Another possibility is that the cochlear dysfunction may require extra cognitive effort and result in increased cognitive load. The cochlear implantation improving cognitive functions based a longitudinal investigation also contributes to this mechanism (Jayakody et al., 2017). This study demonstrated that the hearing ability has been both associated with verbal and non-verbal cognitive assessments; accordingly, overdiagnosis of cognitive decline in our study seems unlikely to occur. In conclusion, auditory and cognitive functions are complementary and interdependent (Wayne and Johnsrude, 2015); the neurobiological bases and the specific mechanism remain to be further investigated.

### Strengths and Limitations

The present study has several strengths. First, this was a prospective study, and it identified the optimal auditory predictor of cognitive impairment based on multiple domains and frequencies of the hearing assessment. Second, although a growing number of studies have documented significant association between hearing loss and cognitive decline, there is no objective audiological test for predicting the risk of the cognitive dysfunction. In this study, H-DPOAE was found to be tightly linked with cognitive domain scores, suggesting that it is a possible objective audiological screening method of individuals at risk of cognitive impairment in the elderly. Third, we divided the hearing indices of each frequency point into low-frequency and high-frequency bands by performing the factor analysis. In addition to the association between peripheral hearing and cognition, we identified the frequency specificity of this association. Fourth, although hearing loss based on diverse auditory assessments has revealed a significant link with cognitive impairment, this is the first study to assess the high-frequency cochlear dysfunction, emphasizing the necessity for early interventions such as hearing aids, etc.

Some limitations should be acknowledged. First, although an association between the high-frequency cochlear dysfunction and specific cognitive decline was identified in this study, causality remains unknown because this was a cross-sectional study. Second, despite our findings in support of the cognitive-ear link, the neurobiological bases for this link remain elusive. The association between the atrophy of specific brain regions and damage degree of the cochlear receptor cell in PC participants is still unknown. Further investigations based on structural brain changes and neuropathological experiments in animals are still needed. Third, the cognitive function was evaluated by a battery of neuropsychological tests. As these tests require responses of patients to instructions, scores may be influenced by hearing impairment. However, the average PTA of the PC group was 35.47 dB HL; it minimally affects face-to-face communication during the cognitive testing particularly tested by a specialist (Gordon-Salant, 2005). Fourth, the sample size of this study was quite small.

## Conclusion

This study has elucidated that the high-frequency cochlear amplifier dysfunction has a direct predictive effect on cognitive decline and greatly influences the cognitive-ear link. Our results highlight the need for high-frequency cochlear function screening for dementia prevention in elderly adults and hearing aids as an early intervention. Further research focusing on the longitudinal association of high-frequency hearing loss and cognitive decline, as well as the impact of auditory interventions on cognition, is needed to confirm our findings.

## Data Availability Statement

The raw data supporting the conclusions of this article will be made available by the authors, without undue reservation.

## Ethics Statement

All protocols for this study were approved by the Shandong University institutional review board (approval no. 2016-KY-059). The patients/participants provided their written informed consent to participate in this study.

## Author Contributions

YW and FG had full access to all of the data in the study and take responsibility for the integrity of the data and the accuracy of the data analysis. YW and FG designed the experiments. XL and WM carried out the experiments. ZQ, SL, and YZ analyzed the experimental results. YW and FG wrote the manuscript. All authors contributed to the article and approved the submitted version.

## Conflict of Interest

The authors declare that the research was conducted in the absence of any commercial or financial relationships that could be construed as a potential conflict of interest.

## Publisher’s Note

All claims expressed in this article are solely those of the authors and do not necessarily represent those of their affiliated organizations, or those of the publisher, the editors and the reviewers. Any product that may be evaluated in this article, or claim that may be made by its manufacturer, is not guaranteed or endorsed by the publisher.
