# Supplementary material for: High-Frequency Cochlear Amplifier Dysfunction: A Dominating Contribution to the Cognitive-Ear Link
Source: Front Aging Neurosci. 2022 Jan 6;13:767570. doi: 10.3389/fnagi.2021.767570 (PMC8770931; doi:10.3389/fnagi.2021.767570)
Supplement: Supplementary file 1 [file Data_Sheet_1.PDF]

Supplementary Material

High-frequency Cochlear Amplifier Dysfunction: a Dominating Contribution to the Cognitive-ear Link

Yao Wang†, Xiao Li†, Fuxin Ren, Siqi Liu, Wen Ma, Yue Zhang, Zhihang Qi, Jing Yang, Honghao Li, Xinxing Fu, Huiquan Wang* and Fei Gao*

†These authors have contributed equally to this work and share first authorship.

***These authors have contributed equally to this work and share corresponding authorship.**

Fei Gao
feigao@email.sdu.edu.cn

Huiquan Wang
huiquan@tiangong.edu.cn

Supplementary Table 1. Inclusion and exclusion criteria defined for participants in this study

PATHOLOGY	INCLUSION OR EXCLUSION
**AUDIOLOGICAL AND CLINICAL OTOLOGICAL PATHOLOGIES**	
Ear disease other than presbycusis	Exclude
Subjects with cochlear implant	Exclude
1) Clinical criteria	
a. One or both tympanic membranes scored as acute otitis media, inactive chronic otitis media or active chronic otitis	Exclude
b. Syndromic features of congenital otological abnormalities	Exclude
2) Sensorineural hearing impairment	
a. Menière disease	Exclude
b. VIII nerve tumor	Exclude
c. Ramsay hunt syndrome	Exclude
d. Post-meningitis	Exclude
e. All sudden losses	Exclude
f. Other genetic hearing loss	Exclude
3) Audiological criteria	
a. Conductive hearing loss, air-bone gap averaged over 0.5,1,2 kHz of >15 dB in one or both ears	Exclude
b. Assymmetrical hearing loss, difference between left and right ear air conduction thresholds of ≥ 20dB for at least 2 frequencies out of 0.5, 1, and 2 kHz	Exclude
**GENERAL PATHOLOGIES**	
1) Cardiovascular disease	
a. Hypertension	
I. Primary	Include
II. Adrenal hyperplasia	Include
b. Hypercholesterolemia	Include, but note if high or low
2) Diabetes	
a. Type I	Include, but carefully write down duration and therapy
b. Type II	
3) Neoplasms	Exclude
4) Neurological disease	Exclude
a. (Alzheimer) dementia	Exclude
b. Parkinson disease	Exclude
c. Multiple sclerosis	Exclude
d. Epilepsy	Exclude
e. Migraine	Exclude
f. Other	Exclude
5) Psychiatric disease	Exclude
6) Other subjects to exclude	
a. All congenital syndromes (Down's syndrome etc.)	Exclude
b. All rare diseases that are severe enough to cause significant handicap according to the subject	Exclude

Supplementary Table 2. The component weights of the matrix after rotation

Frequency(kHz)	Component 1	Component 2
0.125	0.880	0.208
0.25	0.926	0.271
0.5	0.855	0.395
1	0.701	0.516
2	0.435	0.800
4	0.211	0.920
8	0.293	0.848

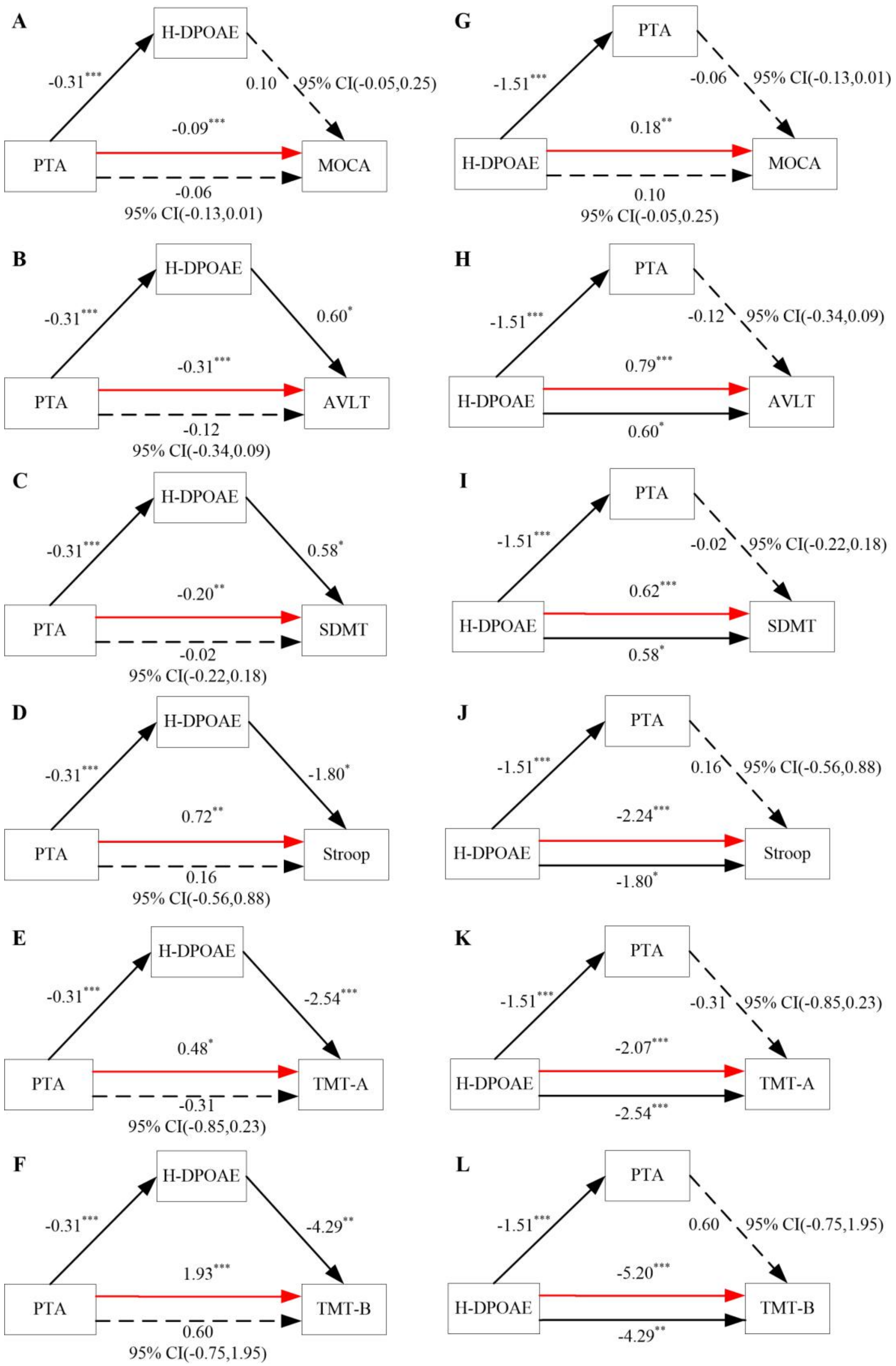

Supplementary Figure 1. Mediating analysis models of H-DPOAE and PTA on cognition in all participants.Left column shows mediation effect characteristics of H-DPOAE between PTA and cognitive tests (A~F), and right column shows mediation effect characteristics of PTA between H-DPOAE and cognitive tests (G~L). Unstandardized regression coefficients and confidence intervals are shown for each path ($p < 0.05^{*}$, $p < 0.01^{**}$, $p < 0.001^{***}$). The solid line represents the path's significance, and the dashed line represents the path's insignificance. Red lines indicate total effect of the independent variables on dependent variables. The 95% CI indicate the confidence interval or range of values across which β would be expected to occur 95% of the time.

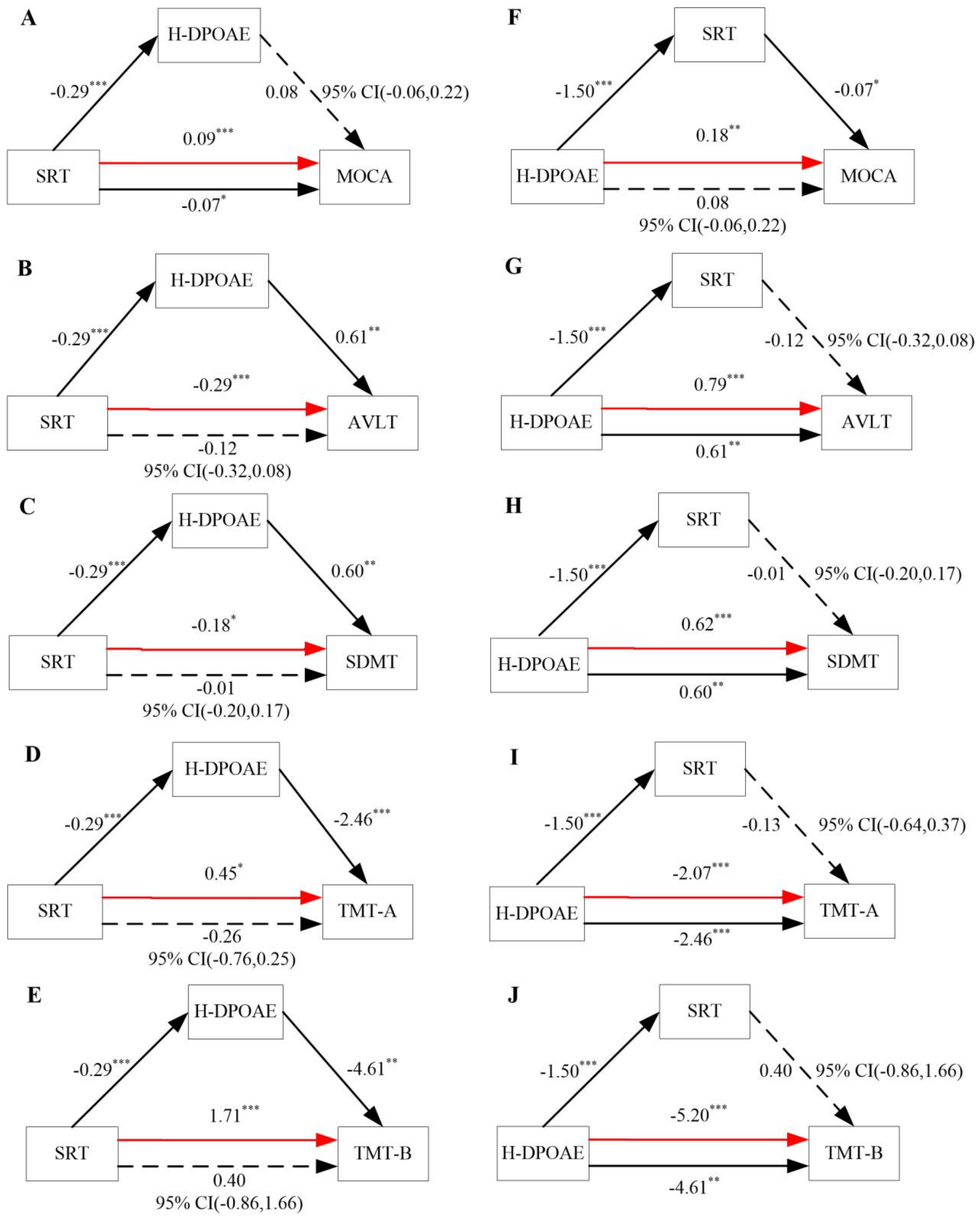

Supplementary Figure 2. Mediating analysis models of H-DPOAE and SRT on cognition in all participants.Left column shows mediation effect characteristics of H-DPOAE between SRT and cognitive tests (A~E), and right column shows mediation effect characteristics of SRT between H-DPOAE and cognitive tests (F~J). Unstandardized regression coefficients and confidence intervals are shown for each path ($p < 0.05^{*}$, $p < 0.01^{**}$, $p < 0.001^{***}$). The solid line represents the path's significance, and the dashed line represents the path's insignificance. Red lines indicate gross effect of the independent variables on dependent variables. The 95% CI indicate the confidence interval or range of values across which β would be expected to occur 95% of the time.
